# Adoption of New Risk Stratification Technologies Within US Hospital Referral Regions and Association With Prostate Cancer Management

**DOI:** 10.1001/jamanetworkopen.2021.28646

**Published:** 2021-10-08

**Authors:** Michael S. Leapman, Rong Wang, Henry S. Park, James B. Yu, Preston C. Sprenkle, Michaela A. Dinan, Xiaomei Ma, Cary P. Gross

**Affiliations:** 1 Department of Urology, Yale School of Medicine, New Haven, Connecticut; 2 Yale Cancer Outcomes, Public Policy, and Effectiveness Research Center, New Haven, Connecticut; 3 Department of Chronic Disease Epidemiology, Yale School of Public Health, New Haven, Connecticut; 4 Department of Therapeutic Radiology, Yale School of Medicine, New Haven, Connecticut; 5 Department of Internal Medicine, Yale School of Medicine, New Haven, Connecticut

## Abstract

**Importance:**

The clinical decisions that arise from prostate magnetic resonance imaging (MRI) and genomic testing in patients with prostate cancer are not well understood.

**Objective:**

To evaluate the association between regional uptake of prostate MRI and genomic testing and observation vs treatment for prostate cancer.

**Design, Setting, and Participants:**

This retrospective cohort study of commercial insurance claims for prostate MRI and genomic testing included 65 530 patients 40 to 89 years of age newly diagnosed with prostate cancer from July 1, 2012, through June 30, 2019.

**Exposures:**

Patient- and regional-level use of prostate MRI and genomic testing.

**Main Outcomes and Measures:**

Observation vs definitive treatment for prostate cancer. Patient-level analyses examined the association between receipt of testing or residing in a hospital referral region (HRR) that adopted testing and observation. In regional-level analyses, the dependent variable was the change in the proportion of patients observed for prostate cancer at the HRR level between 2 periods: July 1, 2012, to June 30, 2014, and July 1, 2017, to June 20, 2019. The independent study variables included HRR-level changes in the proportion of men undergoing prostate MRI and genomic testing between these periods, and the models were adjusted for contextual factors associated with prostate cancer care and socioeconomic status.

**Results:**

This study identified 65 530 patients, including 27 679 in the early period (mean [SD] age, 58.0 [5.9] years) and 37 851 in the late period (mean [SD] age, 59.0 [5.7] years). Use of prostate MRI increased significantly from 7.2% (95% CI, 6.9%-7.5%) to 16.7% (95% CI, 16.3%-17.1%) from the early to late period. Use of genomic testing increased significantly from 1.3% (95% CI, 1.1%-1.4%) to 12.7% (95% CI, 12.3%-13.0%) from the early to late period. Compared with the lowest, the highest HRR quartiles of prostate MRI and genomic testing uptake were associated with an adjusted 4.1% (SE, 1.1%; *P* < .001) and 2.5% (SE, 1.1%; *P* = .03) absolute increase in the proportion of patients receiving observation, respectively.

**Conclusions and Relevance:**

In this cohort study, uptake of prostate MRI and genomic testing was associated with increased use of initial observation vs treatment for prostate cancer. Marked geographic variation supports the need for further patient-level research to optimize the dissemination and outcome of testing.

## Key Points

**Question:**

Is adoption of prostate magnetic resonance imaging (MRI) and genomic testing associated with increased use of observation rather than active treatment for prostate cancer?

**Findings:**

In this cohort study of 65 530 commercially insured patients with prostate cancer, uptake of prostate MRI and genomic testing was associated with increased use of observation vs active treatment as initial management. Although prostate MRI, genomic testing, and observation increased overall, use was highly varied across hospital referral regions.

**Meaning:**

The findings of this study suggest that adoption of technologies designed to improve decision-making may lead to perceivable reductions in overtreatment of prostate cancer

## Teaser

This cohort study assesses the association between the use of prostate magnetic resonance imaging and genomic testing and the initial management of prostate cancer.

## Introduction

There have been dramatic recent increases in the acceptance of active surveillance for prostate cancer, a period of close monitoring followed by timely treatment if needed.^1^ Nonetheless, nearly half of eligible patients still receive immediate active treatment, leading to preventable toxic effects and expense.^1,2^ Magnetic resonance imaging (MRI) of the prostate and several gene expression panels (genomic tests) have been developed and clinically implemented to improve decision-making for localized prostate cancer. Prostate MRI is a staging and diagnostic tool that enhances estimates of cancer grade and stage, whereas genomic tests provide prognostic estimates that are derived from observed associations with disease outcome.^3,4,5,6^ By clarifying the prognosis of screening-detected prostate cancers, it has been assumed that these tools will likely improve the precision of management and increasingly support observational management such as active surveillance.^7^

Despite rapid diffusion into clinical practice, no randomized clinical trials or observational studies have, to our knowledge, addressed the effects of these tests on the initial management of prostate cancer at the population level. Prostate MRI improves the diagnosis and staging of prostate cancer, and clinical guidelines increasingly endorse MRI to improve risk stratification when considering observational management.^8,9,10^ However, little is known about the decisions that arise from real-world use. Retrospective and registry-based studies suggest that genomic tests augment decision-making, steering more patients toward observation, but are limited by their study design and possible external bias from industry sponsorship.^11,12,13^ Determining the effectiveness of these technologies is critical given their expense and potential for decisional conflict associated with their results.^14^ The availability of these technologies also coincides with transformational shifts in the acceptance of observation management strategies, such as active surveillance.^10^ Thus, the extent to which prostate MRI and genomic testing have directly facilitated the adoption of observation rather than immediate treatment may be difficult to appreciate in analyses conducted at the individual patient level.

We assessed the association between the use of prostate MRI and genomic testing and the initial management of prostate cancer. Conventional methods for assessing causal inference using administrative claims are prone to bias because of unmeasured clinical and pathological factors and the subtleties of preference.^15^ As a result, even rigorous adjustment at the patient level may fail to counter the effects of unmeasured confounding and reverse causality. One method to overcome these constraints is to perform analyses across groups of patients who reside in different regions, taking advantage of well-known regional variation in health care.^16,17^ That is, if testing is associated with the decision to observe prostate cancer, we would expect overall increasing use of observation in areas where testing has increased. In this analysis, we use complementary approaches aimed at triangulating effect estimates conducted at the patient and regional levels.

## Methods

### Study Design and Cohort Selection

We performed a retrospective cohort study, with analysis conducted at the patient and hospital referral region (HRR) levels, using geographic boundaries described in the Dartmouth Atlas of Healthcare.^18,19^ The primary data source was deidentified claims data from Blue Cross Blue Shield Axis, a federation of 36 individual health insurance organizations and companies that provides care to approximately one-third of all Americans. Using a secure data portal, we accessed deidentified, longitudinally linked claims from Blue Cross Blue Shield Axis. We included patients 40 to 89 years of age who were newly diagnosed with prostate cancer from July 1, 2012, through June 30, 2019. To ensure that we captured newly diagnosed, incident prostate cancers, we required that the diagnosis coincided with a claim for prostate biopsy within 90 days and that patients had been enrolled in Blue Cross Blue Shield Axis for at least 12 months before the date of prostate cancer diagnosis.

The second source was publicly available data from the Dartmouth Atlas Project, a population-based, small area analysis of health care contextual factors, expenditures, services, and outcomes.^20^ We excluded HRRs that diagnosed fewer than 20 patients in any of the two 24**-**month period of interest (July 1, 2012, to June 30, 2014, and July 1, 2017, to June 30, 2019). The study schema is presented in eFigure 1 in the Supplement. This study was deemed non–human participant research by the Yale University Institutional Review Board, and the need for informed consent was therefore waived. This study followed the Strengthening the Reporting of Observational Studies in Epidemiology (STROBE) reporting guideline (eAppendix in the Supplement).

### Study Variables

#### Patient-Level Measures

We identified claims for prostate MRI and commercial genomic testing surrounding a patient’s initial diagnosis of prostate cancer. For prostate MRI, we included claims in the 6 months before and after diagnosis. For genomic tests, we used a previously described algorithm based on *Current Procedural Terminology* codes linked to laboratory-specific National Provider Identifier numbers.^21^ We focused on tests included in the 2020 National Comprehensive Cancer Network’s Prostate Cancer Clinical Guideline and assessed their use in the 6-month period after diagnosis (eAppendix in the Supplement).

We assessed a patient’s treatment status based on claims for definitive surgery, radiotherapy, androgen deprivation, or focal therapy in the 6-month period after diagnosis based on *Current Procedural Terminology* codes using previously applied algorithms.^22^ Patients without definitive treatment within 6 months of their prostate cancer diagnosis were regarded as undergoing observation.

#### Regional-Level Measures

We assigned patients to HRR boundaries based on billing zip code. We calculated the proportion of patients within each HRR who received genomic testing, prostate MRI, and initial definitive treatment or observation. We determined HRR-level proportions of genomic testing, prostate MRI, and treatment for prostate cancer in the following 2 periods: July 1, 2012, to June 30, 2014 (early), and July 1, 2017, to June 30, 2019 (late). These periods were selected to reflect an early period before widespread availability of these technologies and a later period characterized by wider interest and availability. The intervening July 1, 2014, to June 30, 2017, period was used as a washout. To account for differences in baseline use that existed between regions, we further calculated the absolute change in these measures (genomic testing, MRI, and observation) between the early and late periods and stratified the magnitude of HRR-level change by quartile. We compiled health care contextual measures associated with prostate cancer care that were described at the HRR level in 2014.^20^

### Statistical Analysis

We compared sociodemographic characteristics of HRRs across quartiles of prostate MRI and genomic testing change between the early and late study periods. We assessed the association between change in prostate MRI and genomic testing at the HRR level using frequency tables and Spearman rank correlation coefficient. We used ordinary least squares regression to study the association between the quartile of HRR-level change in the use of prostate MRI and genomic testing and changes in the proportion of patients with prostate cancer undergoing observation. The regression models were weighted by the number of patients in each HRR in the early period. The dependent variable was the change in the proportion of patients observed for prostate cancer at the HRR level between 2 periods: July 1, 2012, to June 30, 2014, and July 1, 2017, to June 30, 2019. The independent study variables included the change in HRR-level use of genomic testing, prostate MRI, and contextual factors of prostate cancer care.

In a secondary analysis conducted at the patient level, we examined the association between prostate MRI and genomic testing and observation for prostate cancer using descriptive statistics. We constructed 2 series of multivariable logistic regression models to estimate the patient-level associations of receiving testing and residing in an HRR that adopted testing to varying degrees. The dependent variable in both models was observation vs treatment for prostate cancer. We first constructed a mixed-effects logistic regression model that incorporated an HRR random intercept to account for clustering of patients within regions with distinct patterns of prostate cancer care. The independent variables were the patient status of prostate MRI and genomic testing. The models were adjusted for age and HRR-level contextual factors (median household income and historical rates of prostate-specific antigen screening). In separate mixed-effects model restricted to the early and late periods, the independent variables included the quartile rank of prostate MRI and genomic testing change between the early and late periods. The model was similarly adjusted for age, diagnosis year, and HRR-level contextual factors. A 2-sided *P* < .05 was considered to be statistically significant. Statistical analyses were performed in SAS software, version 9.4 (SAS Institute Inc).

## Results

### Patient-Level Analysis

A total of 65 530 patients were identified, including 27 679 in the early period (mean [SD] age, 58.0 [5.9] years) and 37 851 in the late period (mean [SD] age, 59.0 [5.7] years). Eligible patients with prostate cancer were drawn from 236 HRRs that contributed more than 20 patients in each evaluation period. The proportion of patients who received prostate MRI increased from 7.2% (95% CI, 6.9%-7.5%) to 16.7% (95% CI, 16.3%-17.1%) from the early to late period; the proportion of patients who received genomic testing increased from 1.3% (95% CI, 1.1%-1.4%) in the early period to 12.7% (95% CI, 12.3%-13.0%) in late period (Figure). A total of 64 377 patients (98.2%) who received testing did so with a single modality; 1153 (3.0%; 95% CI, 2.9%-3.2%) received both prostate MRI and genomic testing in the late study period (Table 1).

**Figure.**
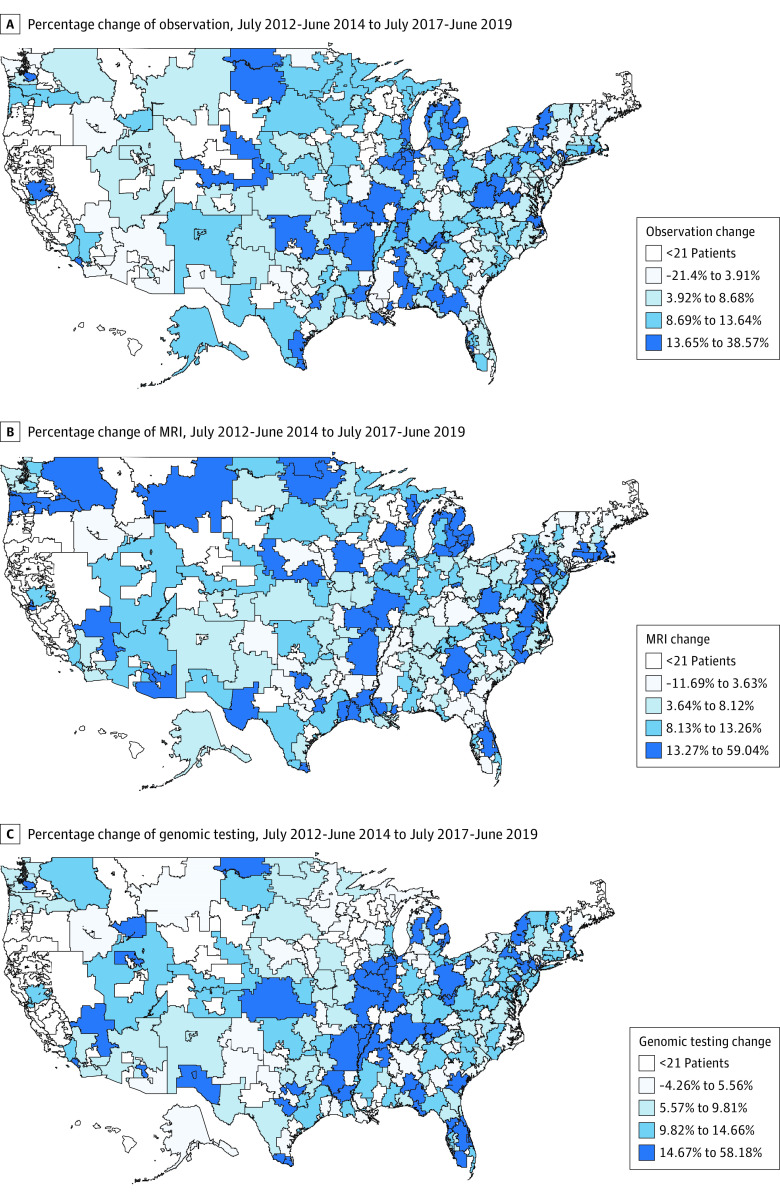
Changes in Hospital Referral Region–Level Use of Prostate Magnetic Resonance Imaging (MRI), Genomic Testing, and Observation for Newly Diagnosed Prostate Cancer Stratified by Quartile, July 2012-June 2014 to July 2017-June 2019

**Table 1.** Characteristics of the Study Cohort at the Patient Level by Study Period^a^

Characteristic	Study period	
	July 1, 2012, to June 30, 2014 (n = 27 679)	July 1, 2017, to June 30, 2019 (n = 37 851)
Age at diagnosis, mean (SD), y	58 (5.9)	59 (5.7)
Year of diagnosis		
2012-2013	13 950 (50.4)	NA
2013-2014	13 729 (49.6)	NA
2017-2018	NA	19 175 (50.7)
2018-2019	NA	18 676 (49.3)
Received		
Prostate MRI	2001 (7.2)	6325 (16.7)
Genomic testing	347 (1.3)	4801 (12.7)
Both prostate MRI and genomic testing	69 (0.2)	1153 (3.0)
Management		
Observation	7312 (26.4)	13 408 (35.4)
Radiotherapy	5070 (18.3)	5092 (13.5)
Radical prostatectomy	12 871 (46.5)	15 490 (40.9)
Other (including androgen deprivation therapy)	2426 (8.8)	3861 (10.2)
Income in the HRR, mean (SD), $^c^	582 90.00 (13 187.2)	58 732.2 (13 477.5)
PSA testing among men 68-74 y of age in the HRR, mean (SD), %^c^	33.4 (10.7)	33.9 (10.6)

Abbreviations: HRR, hospital referral region; MRI, magnetic resonance imaging; NA, not applicable; PSA, prostate-specific antigen.

^a^ Data are presented as number (percentage) of patients unless otherwise indicated.

^b^ Overlap in year is due to time periods being from July 1 to June 30.

^c^ Contextual measures from Dartmouth Health Atlas; not measured at the patient level.

The proportion of patients whose cases were managed with observation increased from 26.4% (95% CI, 25.9%-26.9%) in the early period to 35.4% (95% CI, 34.9%-35.9%) in the late period (Table 1). Among patients who underwent prostate MRI in the early period, 40.7% (95% CI, 38.6%-42.9%) were observed compared with 25.3% (95% CI 24.8%-25.8%) among those without prostate MRI. In the late period, observation increased to 52.2% (95% CI, 51.0%-53.4%) among patients who received prostate MRI compared with 32.1% (95% CI, 31.5%-32.6%) among patients who had not received an MRI. Similarly, observation was more common among patients who received genomic testing. In the late period, 59.2% (95% CI, 57.8%-60.6%) of patients who received genomic testing were observed compared with 32.0% (95% CI 31.5%-32.5%) of those without genomic testing.

At the patient level, prostate MRI (odds ratio [OR], 2.18; 95% CI, 2.06-2.30) and genomic testing (OR, 3.05; 95% CI, 2.86-3.25) were associated with increased odds of observation. Residence within an HRR with a higher median household income (OR per $10 000 increase, 1.06; 95% CI, 1.03-1.09) was associated with increased odds of observation (eTable 1 in the Supplement). Table 2 gives the results of mixed-effects logistic regression models examining the effects of HRR-level changes in prostate MRI and genomic testing. Residence in a region that subsequently adopted prostate MRI or genomic testing was not associated with increased odds of observation in the early period. Among patients diagnosed in the late period, residence in an HRR with the highest quartile increase in prostate MRI use (OR, 1.21; 95% CI, 1.09-1.34) and greater adoption of genomic testing (third vs first quartile: OR, 1.14; 95% CI, 1.02-1.27) were associated with increased odds of observation (Table 2).

**Table 2.** Patient-Level Mixed-Effects Logistic Regression of the Association Between Regional Use of Prostate MRI and Genomic Testing and Observation for Prostate Cancer Among Patients Diagnosed in the Early and Late Study Periods

Variable	Diagnosed in early period (July 1, 2012, to June 30, 2014)		Diagnosed in late period (July 1, 2017, to June 1, 2019)	
	Odds ratio (95% CI)	*P* value	Odds ratio (95% CI)	*P* value
Age at diagnosis	1.02 (1.01-1.02)	<.001	1.00 (1.00-1.00)	.35
Change in HRR-level use of prostate MRI				
First quartile	1 [Reference]	NA	1 [Reference]	NA
Second quartile	0.97 (0.85-1.11)	.63	1.05 (0.95-1.17)	.33
Third quartile	0.91 (0.80-1.05)	.19	1.06 (0.96-1.18)	.24
Fourth quartile	1.04 (0.91-1.18)	.59	1.21 (1.09-1.34)	<.001
Change in HRR-level use of genomic testing				
First quartile	1 [Reference]	NA	1 [Reference]	NA
Second quartile	0.98 (0.86-1.12)	.77	1.01 (0.91-1.12)	.92
Third quartile	1.13 (0.98-1.30)	.09	1.14 (1.02-1.27)	.02
Fourth quartile	0.95 (0.83-1.09)	.46	1.11 (1.00-1.23)	.06
Median household income in the HRR (per $10 000)^a^	1.10 (1.06-1.15)	<.001	1.07 (1.04-1.10)	<.001
Patients receiving PSA testing within HRR^a^	1.00 (1.00-1.00)	.77	1.00 (1.00-1.00)	.34

Abbreviations: HRR, hospital referral region; MRI, magnetic resonance imaging; NA, not applicable; PSA, prostate-specific antigen.

^a^ Contextual measures from Dartmouth Health Atlas; not measured at the patient level.

#### Regional-Level Analysis

Contextual population demographic characteristics from the Dartmouth Atlas differed across levels of prostate MRI and genomic testing uptake. The HRRs with greater prostate MRI uptake had lower proportions of Black patients (mean [SD] prostate MRI change quartile, 12.6% [10.2%] in the highest quartile vs 17.3% [13.6%] in the lowest quartile; *P* = 0.03 for trend). The HRRs with greater genomic testing uptake had higher levels of college education (mean [SD], 26.9% [8.4%] in the highest quartile vs 25.0% [5.7%] in the lowest quartile; *P* <.001 for trend) and median income level (mean [SD], $54 286 [$14 248] in the highest quartile vs $51 778 [$9732] in the lowest quartile; *P* = 0.02 for trend). In addition, contextual prostate cancer care patterns differed across quartiles of HRR-level genomic testing uptake. Regions with greater adoption of genomic testing had historically less use of primary androgen deprivation therapy and less use of prostatectomy (Table 3).

**Table 3.** Characteristics of HRRs by Quartile of Change in Prostate MRI and Genomic Testing During the Study Period

Characteristic	Mean (SD) change in HRR-level use				*P* value^a^
	Quartile 1 (lowest)	Quartile 2	Quartile 3	Quartile 4 (highest)	
**Prostate MRI**					
Race or ethnicity, %^a^					
Black	17.3 (13.6)	12.9 (10.8)	11.5 (9.0)	12.6 (10.2)	.03
White	77.6 (14.7)	80.5 (12.2)	81.2 (10.3)	81.7 (10.9)	.26
Other^b^	5.2 (5.4)	6.6 (6.7)	7.3 (7.1)	5.7 (5.2)	.24
College and above, %^a^	26.8 (7.1)	27.7 (7.5)	28.5 (8.4)	27.9 (7.4)	.70
Income, $^a^	52 180.10 (11 950.50)	55 118.40 (13 378.10)	57 144.80 (13 835.00)	55 760.30 (11 385.40)	.19
Urologist density (urologists per 100 000 population) ^a^	2.7 (0.6)	2.6 (0.6)	2.5 (0.4)	2.5 (0.5)	.07
PSA testing among men 68-74 y of age^a^	34.1 (11.6)	31.5 (10.9)	32.6 (10.8)	31.2 (11.6)	.51
Prostate cancer incidence (per 1000 population)^a^	7.5 (2.9)	7.3 (3.1)	7.9 (3.7)	7.6 (4.2)	.81
Use of ADT among men >75 y of age (per 1000 population)^a^	364.5 (86.2)	388.9 (105.4)	377.2 (82.0)	362.4 (77.3)	.37
No treatment of prostate cancer in men >75 y of age (per 1000 population)^a^	357.5 (90.7)	350.0 (92.1)	327.8 (80.4)	362.6 (89.1)	.18
Use of radiotherapy in patients >75 y of age (per 1000 population)^a^	277.3 (81.1)	256.1 (72.7)	271.6 (60.7)	255.5 (59.9)	.31
Use of prostatectomy in patients <75 y of age (per 1000 population)^a^	198.6 (104.2)	186.9 (60.4)	215.3 (77.4)	197.4 (71.7)	.37
**Genomic testing**					
Race or ethnicity, %^a^					
Black	13.5 (12.1)	11.8 (10.3)	16.0 (10.8)	13.0 (11.3)	.23
White	82.1 (12.0)	81.1 (11.3)	76.4 (12.7)	81.4 (12.1)	.04
Other^b^	4.4 (4.4)	7.0 (6.6)	7.7 (6.9)	5.7 (6.0)	.02
College and above, %^a^	25.0 (5.7)	28.3 (7.2)	30.8 (7.6)	26.9 (8.4)	<.001
Income, $^a^	51 778.3 (9732.20)	54 952.6 (10 843.00)	59 186.3 (14 559.60)	54 286.3 (14 248.50)	.02
Urologist density (urologists per 100 000 population)^a^	2.6 (0.5)	2.4 (0.4)	2.6 (0.5)	2.5 (0.6)	.16
PSA testing among men 68-74 y of age^a^	30.3 (10.0)	31.5 (11.7)	33.9 (11.6)	33.6 (11.4)	.24
Prostate cancer incidence (per 1000 population)^a^	7.8 (3.4)	7.4 (3.8)	7.1 (2.4)	8.0 (4.1)	.51
Use of ADT among men >75 y of age (per 1000 population)^a^	412.1 (111.0)	356.4 (71.0)	362.9 (75.5)	361.3 (80.0)	.002
No treatment of prostate cancer in men >75 y of age (per 1000 population)^a^	335.0 (85.5)	356.9 (85.1)	359.1 (81.5)	343.7 (100.9)	.47
Use of radiotherapy in patients >75 y of age (per 1000 population)^a^	271.3 (70.2)	261.2 (68.2)	263.6 (65.4)	266.4 (74.4)	.91
Use of prostatectomy in patients <75 y of age (per 1000 population)^a^	228.9 (83.2)	200.8 (85.7)	169.1 (64.7)	201.9 (75.5)	.003

Abbreviations: ADT, androgen deprivation therapy; HRR, hospital referral region; MRI, magnetic resonance imaging; PSA, prostate-specific antigen.

^a^ Contextual measures from Dartmouth Health Atlas; not measured at the patient level.

^b^ Other race or ethnicity includes all races or ethnicities other than Black or White.

Among the 236 HRRs that contributed more than 20 patients in each evaluation period, substantial regional variation in testing occurred. At the HRR level, use of prostate MRI in the late study period ranged from 0% to 62.7%, and the use of genomic testing ranged from 0% to 58.2%. Similarly, the initial management approach varied substantially, with observation ranging from 15.6% to 58.3% across HRRs. The mean (SD) change over time in HRR-level use was 9.0% (8.0%) for prostate MRI, 11.0% (8.2%) for genomic testing, and 9.0% (9.3%) for observation. Increases in HRR-level use of prostate MRI testing (ρ = 0.23, *P* < .001) and genomic testing (ρ = 0.18, *P* = .005) were positively correlated with change in observation (eFigure 2 in the Supplement). In multivariable linear regression, mean (SE) increases in the proportion of observed patients of 4.09% (1.06%) (*P* < .001) for the highest quartile (vs lowest) of prostate MRI and 2.47% (1.11%) (*P* = .03) for the highest quartile (vs lowest) of genomic testing uptake were observed. No significant association was seen between the mean (SE) second or third quartile rank of prostate MRI (mean [SE], 1.00 [1.03] in the second quartile [*P* < .001] vs 1.09 [1.07] in the third quartile [*P* < .001]) and genomic testing uptake (0.06 [1.05] in the second quartile [*P* = .96] vs 2.01 [1.14] in the third quartile [*P* = .08]). Increases in observation were also associated with baseline observation rate (estimate [SE], –0.71 [0.06]; *P* < .001), contextual median household income (estimate [SE], 1.02 [0.30]; *P* = .001), and prostate-specific antigen testing rates (estimate [SE], –0.07 [0.03]; *P* = .04) (Table 4).

**Table 4.** Results of Weighted Multivariable Linear Regression on the Associations of HRR-Level Adoption of Prostate MRI and Genomic Testing and Observation for Prostate Cancer Among 236 HRRs

Variable	Estimate (SE)	*P* value
Intercept	21.17 (2.20)	<.001
Change in HRR-level use of prostate MRI		
First quartile (lowest)	1.00	<.001
Second quartile	1.00 (1.03)	.33
Third quartile	1.09 (1.07)	.31
Fourth quartile (highest)	4.09 (1.06)	<.001
Change in HRR-level use of genomic testing		.03
First quartile (lowest)	1.00	
Second quartile	0.06 (1.05)	.96
Third quartile	2.01 (1.14)	.08
Fourth quartile (highest)	2.47 (1.11)	.03
Baseline observation	–0.71 (0.06)	<.001
Median household income in the HRR (per $10 000)	1.02 (0.30)	.001
PSA testing	–0.07 (0.03)	.04

Abbreviations: HRR, hospital referral region; MRI, magnetic resonance imaging; PSA, prostate-specific antigen.

## Discussion

This cohort study found substantial increases in the use of prostate MRI and prognostic tissue–based genomic testing among commercially insured men with prostate cancer between July 2012 and June 2019. During the same period, the overall proportion of men who were initially observed, rather than treated, for prostate cancer also increased substantively. Despite increases in these practices overall, changes in prostate MRI, genomic testing, and observation varied widely across geographic regions. Examining the association between testing and initial management at the patient and regional levels, we found that uptake of prostate MRI and genomic testing was associated with increases in the use of observation. However, these associations were limited to HRRs with greater uptake of prostate MRI and genomic testing. In the context of an expanding array of new diagnostic and prognostic tools in cancer care, these findings can guide discussions about the clinical decisions associated with their use. Prominent regional variation in the use of risk stratification technologies also emphasizes an opportunity to address emerging gulfs in utilization, particularly as they may relate to overtreatment.

Distinct prostate cancer treatment practices among high-use regions may reflect several coexisting factors. The technologies themselves may exert direct effects as anticipated, leading to changes in management within an HRR that were perceivable only at high levels. Such an explanation would be congruent with analyses conducted at the patient level and prior evidence sources for prostate MRI and genomic testing, drawing the association between the use of refined risk assessment tools and greater use of observation for prostate cancer.^23,24^ It is also likely that regions with greater uptake of emerging diagnostic staging and risk assessment technologies shared conditions that favored greater increases in observation but did not directly affect the decision for treatment or observation, such as a region’s health care infrastructure and culture.^25,26^ However, we found that adoption of these technologies did not necessarily occur in HRRs with greater baseline inclinations for observation in the period before more widespread availability of these tools. Future research to better understand the relative contribution of these influences is warranted given the mixed track record of several other diagnostic technologies in oncology, including many that rapidly enter standard clinical care.^27,28,29,30^

Associations between regional expansion of new risk stratification technologies and observation for prostate cancer should be viewed in the context of global shifts in support of active surveillance. During the period in which prostate MRI and genomic testing became clinically integrated, practice guidelines also became explicit in their support for active surveillance as the preferred management strategy for most patients with low-risk prostate cancer, consolidating years of research and advocacy.^31,32^ Although adjunctive tests, such as prostate MRI and genomic testing, are not required to select patients for active surveillance, evidence supporting their role in refining risk and reducing diagnostic uncertainty has flourished.^33^ Indeed, we observed significant increases in the overall and HRR-level use of the technologies in line with prior estimates^10,34,35^ Associations between high HRR-level use and changes in management further imply a role for these technologies within common pathways for observation. However, the strength of the linear associations is weak, and the effect estimates were quite modest. Attenuation of the effect estimates when conducted at the regional vs patient level are also notable and can serve a methodologic caveat when performing effectiveness research using administrative claims. As a result, our results should be interpreted as an indication of alignment between regional increases in observation and tools aimed to facilitate its application, without causal attribution or directionality.

### Limitations

This study has limitations. Patient-level analyses are limited by the absence of granular clinical information used in decision-making. Among this undifferentiated sample of men with prostate cancer, use of testing is expected to be confounded by lower-risk status, which may lead to both the exposure (use of testing) and outcome (observation). We suspect that concerns are more prominent for genomic testing, based on firmer indications for favorable-risk disease, but could also affect estimates for prostate MRI. By performing analyses at the regional level, this study is also subject to biases associated with ecologic inference. Therefore, effect estimates must be interpreted at the HRR level and cannot necessarily be extrapolated to individual patients. Similar directions of association in secondary analyses conducted at the patient level are reassuring but not conclusive. By aggregating information within groups of patients, we do not account for individual patient characteristics that might modify the association between the adoption of technologies and observation for prostate cancer. However, patient characteristics explain only a small amount of variation in the use of observational management, which is largely driven by other factors, such as a patient’s geographic region or treating physician and facility.^36^ As a result, our analysis is responsive to external determinants of initial management strategy, which might be modified by the use of prostate MRI or genomic testing. We also adjusted for historical contextual factors at the HRR level described in the Medicare population, which may not be directly applicable to the younger, privately insured cohort. In addition, because we used anonymized administrative claims, we were unable to incorporate the results of testing or the appropriateness of management decisions that arose from their use.^37^

## Conclusion

Although use increased overall, uptake of prostate MRI and genomic testing surrounding prostate cancer diagnosis varied considerably across regional health care marketplaces measured at the HRR level. Similarly, use of initial observation vs active treatment for prostate cancer increased overall, but the magnitude of change varied significantly by HRR. The highest quartile of uptake of prostate MRI and genomic testing was associated with increases in the use of observation for the initial management of prostate cancer. Less rapid adoption of the technologies was not associated with increases in observation for prostate cancer. These findings suggest alignment between a region’s use of new risk stratification techniques occurring at the extremes and changes in the use of observational management for prostate cancer.

## Notes

Supplement. **eFigure 1**. Selection of Study Cohort **eFigure 2.** Scatterplot Depicting the Association Between HRR-Level Changes in the Use of (A) Prostate MRI (B) Genomic Testing and Observation for Prostate Cancer **eAppendix.** Prostate Cancer Genomic Tests Included in Analysis
