# Supplementary material for: Adoption of New Risk Stratification Technologies Within US Hospital Referral Regions and Association With Prostate Cancer Management
Source: JAMA Netw Open. 2021 Oct 8;4(10):e2128646. doi: 10.1001/jamanetworkopen.2021.28646 (PMC8501394; doi:10.1001/jamanetworkopen.2021.28646)
Supplement: Supplement. — eFigure 1. Selection of Study Cohort eFigure 2. Scatterplot Depicting the Association Between HRR-Level Changes in the Use of (A) Prostate MRI (B) Genomic Testing and Observation for Prostate Cancer eAppendix. Prostate Cancer Genomic Tests Included in Analysis [file jamanetwopen-e2128646-s001.pdf]

Supplementary Online Content

Leapman MS, Wang R, Park HS, et al. Adoption of new risk stratification technologies within US hospital referral regions and association with prostate cancer management. *JAMA Netw Open*. 2021;4(10):e2128646. doi:10.1001/jamanetworkopen.2021.28646

eFigure 1. Selection of Study Cohort

eFigure 2. Scatterplot Depicting the Association Between HRR-Level Changes in the Use of (A) Prostate MRI (B) Genomic Testing and Observation for Prostate Cancer

eAppendix. Prostate Cancer Genomic Tests Included in Analysis

This supplementary material has been provided by the authors to give readers additional information about their work.

© 2021 Leapman MS et al. *JAMA Network Open.*

eFigure 1. Selection of Study Cohort

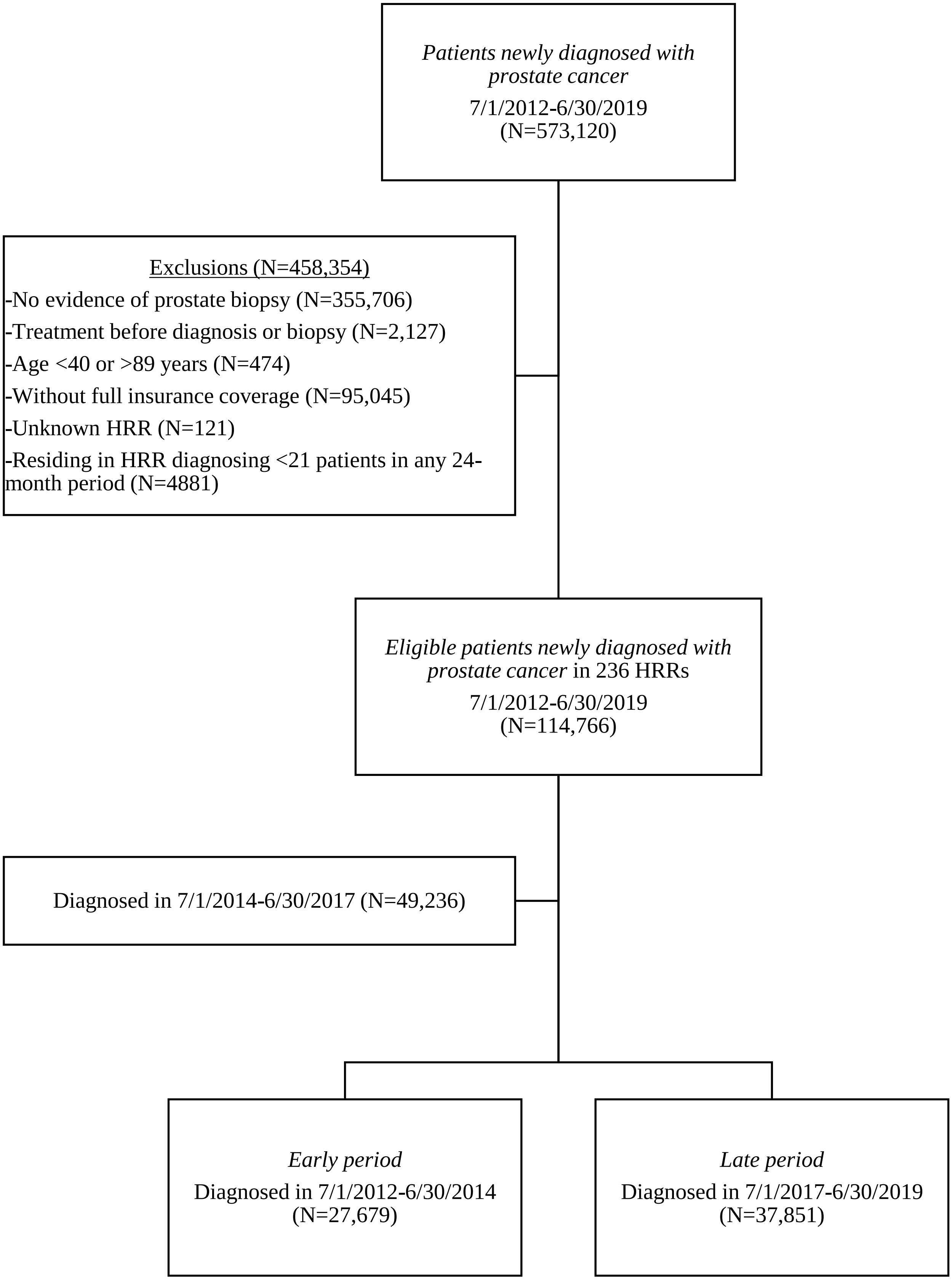

© 2021 Leapman MS et al. *JAMA Network Open.*

eFigure 2. Scatterplot Depicting the Association Between HRR-Level Changes in the Use of (A) Prostate MRI (B) Genomic Testing and Observation for Prostate Cancer

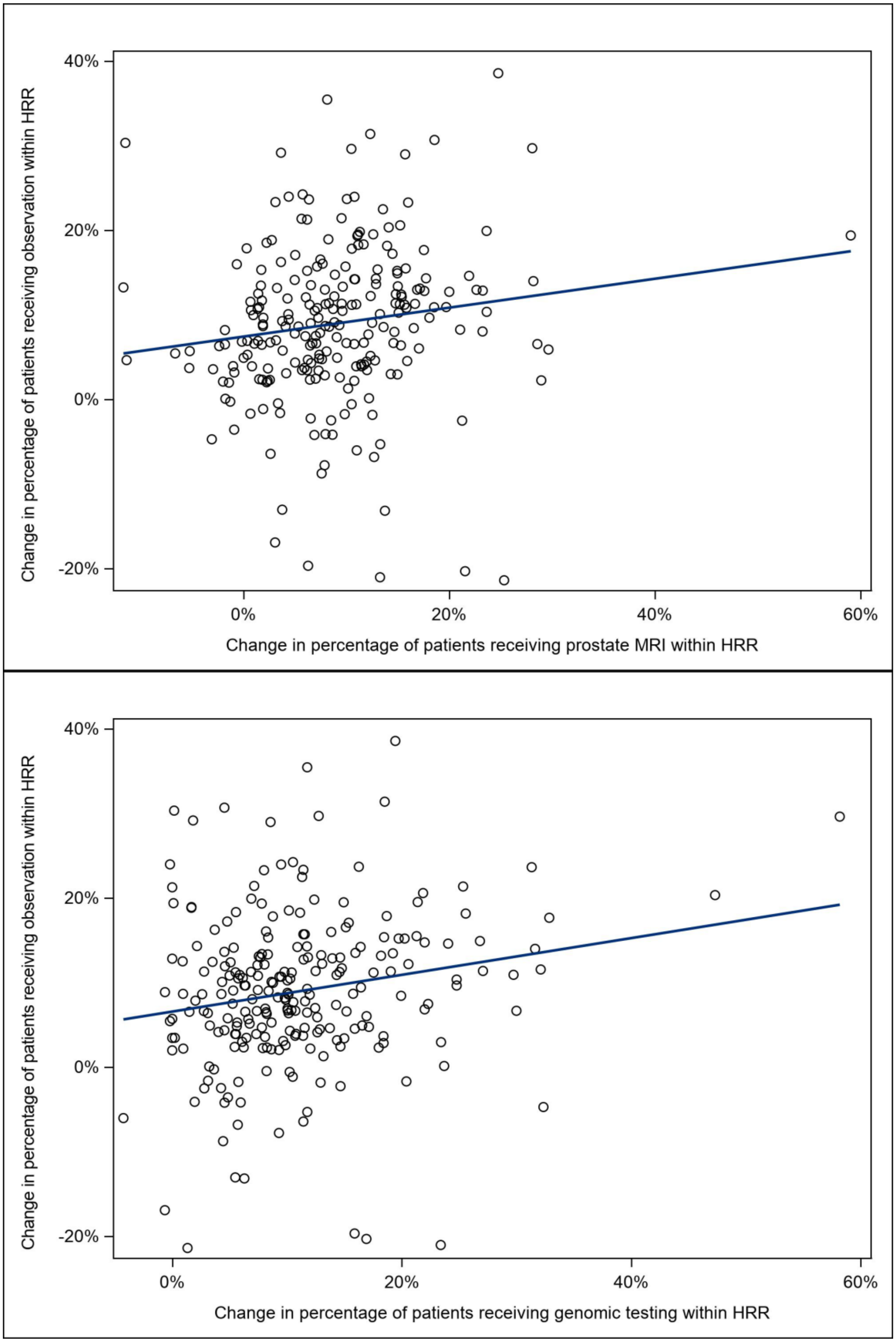

© 2021 Leapman MS et al. *JAMA Network Open.*

eAppendix. Prostate Cancer Genomic Tests Included in Analysis

**Prostate Cancer Assay**	**Manufacturer**
Decipher®	Genome DX, Vancouver BC
OncotypeDX GPS®	Genomic Health, Redwood City, CA
Prolaris ®	Myraid Genetics, Salt Lake City, UT
Promark®	Metamark Genetics, Waltham MA

© 2021 Leapman MS et al. *JAMA Network Open.*
